# Effect of prevention of mother-to-child transmission strategies on antiretroviral therapy coverage in pregnant women in Zambia: analysis using routinely collected data (2010–15)

**DOI:** 10.1016/s2214-109x(19)30110-x

**Published:** 2019-03-08

**Authors:** Sehlulekile Gumede-Moyo, Jim Todd, Ab Schaap, Paul Mee, Suzanne Filteau

**Affiliations:** London School of Hygiene and Tropical Medicine, London, UK (S Gumede-Moyo MPH, J Todd MA, A Schaap MSc, P Mee PhD, S Filteau PhD)

## Abstract

**Background:**

The WHO recommendation for lifelong antiretroviral therapy (ART) for all pregnant and lactating women (Option B+) has been adopted by nearly all countries with the highest HIV burdens. In January, 2013, Zambia announced that it would revise its guidelines on prevention of mother-to-child transmission (PMTCT) and adopt Option B+. Accurate estimates of coverage of PMTCT services in pregnant women with HIV are vital for monitoring progress towards HIV elimination targets. We aimed to show trends in the coverage of PMTCT services in Zambia from 2010 to 2015. We describe the proportion of women attending antenatal care who are HIV positive, the proportion of HIV-positive pregnant women who started ART, the proportion of HIV-infected women already on ART, and time to treatment initiation.

**Methods:**

This was a retrospective cohort study using routinely collected data from SmartCare, an electronic health record system. We included data from all pregnant women who attended antenatal care in the 889 health facilities using the SmartCare database in Zambia.

**Findings:**

We included data for 104 155 pregnant women who attended antenatal services in SmartCare facilities between Jan 1, 2010, and Dec 31, 2015. Of these women, 9% (9262) tested HIV-positive during antenatal visits and 43% (44 387) had missing HIV test results. Almost half (47%, [4375]) the pregnant women who tested HIV-positive in their antenatal visit were recorded in 2010. Among HIV-positive women, there was an increase in those already on ART at first antenatal visit from 9% (40) in 2011 to 74% (1 155) in 2015. In our study, 65% (983/1501) of the women who started ART after testing HIV-positive during antenatal care were documented after the adoption of Option B+ (2013–15). Mean time lag between starting antenatal care and ART initiation was 7 months over the 6 year study period, but there were notable variations between provinces and years.

**Interpretation:**

The implementation of WHO PMTCT guidelines introduced after 2010 in Zambia has resulted in an increase in the proportion of HIV-infected pregnant women attending antenatal care who are already on ART. The SmartCare database could enable Zambian health policy makers to act on urgent PMTCT interventions and improve health-care quality and outcomes for mothers and their infants. However, there is a need first to improve procedures for data collection and entry. The number of missing data observations indicate the need for further qualitative research on why so many records were missing from the database.

**Funding:**

SEARCH (Sustainable Evaluation through Analysis of Routinely Collected HIV data) and the Bill & Melinda Gates Foundation (grant number OPP1084472).

## Declaration of interests

We declare no competing interests.

